# Ultrafast Transient Infrared Spectroscopy of Photoreceptors with Polarizable QM/MM Dynamics

**DOI:** 10.1021/acs.jpcb.1c05753

**Published:** 2021-09-03

**Authors:** Veronica Macaluso, Shaima Hashem, Michele Nottoli, Filippo Lipparini, Lorenzo Cupellini, Benedetta Mennucci

**Affiliations:** Dipartimento di Chimica e Chimica Industriale, University of Pisa, via G. Moruzzi 13, 56124 Pisa, Italy

## Abstract

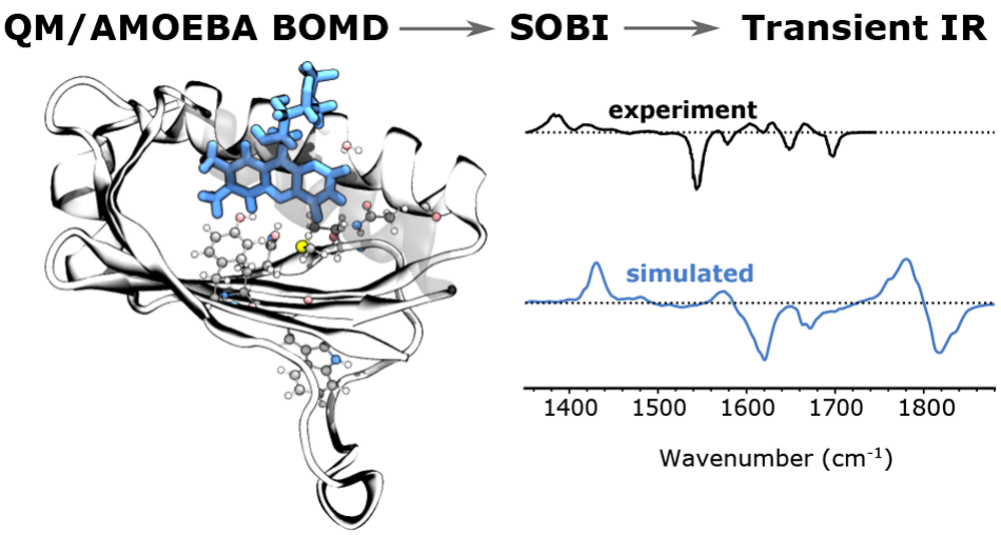

Ultrafast transient infrared (TRIR) spectroscopy is widely used to measure the excitation-induced structural changes of protein-bound chromophores. Here, we design a novel and general strategy to compute TRIR spectra of photoreceptors by combining μs-long MM molecular dynamics with ps-long QM/AMOEBA Born–Oppenheimer molecular dynamics (BOMD) trajectories for both ground and excited electronic states. As a proof of concept, the strategy is here applied to AppA, a blue-light-utilizing flavin (BLUF) protein, found in bacteria. We first analyzed the short-time evolution of the embedded flavin upon excitation revealing that its dynamic Stokes shift is ultrafast and mainly driven by the internal reorganization of the chromophore. A different normal-mode representation was needed to describe ground- and excited-state IR spectra. In this way, we could assign all of the bands observed in the measured transient spectrum. In particular, we could characterize the flavin isoalloxazine-ring region of the spectrum, for which a full and clear description was missing.

## 1 Introduction

Photoreceptors are proteins that use photosensing chromophores to capture light signals from the environment. In recent years, the interest in these systems has largely increased because of their wavelength-range of absorption and the resulting several applications, such as in bioimaging and optogenetics.^1−3^ Unfortunately, these systems are as interesting as they are challenging to be studied and characterized. Substantial progress has been made in the experimental methodologies which may be applied on such systems. For example, X-ray structures of most photoreceptors of interest are nowadays available. The static picture provided by X-ray studies can be enriched by information obtained from solution-based experiments.

Transient IR (TRIR) spectroscopy is a fundamental tool to probe the structure and the environment of protein-bound chromophores. While a direct measurement of the chromophore’s vibrational response is unfeasible due to the overlap of the infrared bands with those of the environment, it is possible to take advantage of the electronic excitations to “select” the chromophore signals over those of the environment. By exciting the chromophore and recording the ultrafast time-resolved IR signals, one obtains information on the vibrational properties of both ground and excited states, and on the time evolution of the chromophore on the excited-state potential energy surface.^4^ Transient IR has thus found wide applications for photoactive proteins,^5^ including blue-light-sensing flavoproteins,^6−11^ phytochromes,^12^ and carotenoid-binding proteins.^13^ With complex chromophores, however, the precise assignment of ground- and excited-state vibrational modes remains challenging. Multiple isotope substitutions can be used to pinpoint the contribution of each atom to the vibrational modes.^8^

A possible improvement in this direction is to combine experiments with computational simulations, which can be used to enrich experimental measurements with atomistic detail. However, the modeling of photoreceptors presents numerous difficulties, which arise from the size of the embedded chromophore itself, the size and dynamics of the protein, and the intricate chromophore–protein relation in both time and space. Multiscale methods, and in particular methods based on quantum mechanics/molecular mechanics (QM/MM), represent the method of choice to study such systems especially when used in combination with molecular dynamics (MD) simulations.^14−20^ Within this framework, QM/MM results need to be averaged over various configurations extracted from the MD trajectories, which are usually performed within a fully classical description using MM force fields. Such a strategy is generally accurate in many cases but can fail for properties deeply and finely connected to the coupling between structural fluctuations of the protein and the chromophore. In fact, while MM MD is usually accurate enough to properly describe the protein dynamics, this is not always the case for the chromophores, especially for the highly conjugated molecules that are involved in photoreceptors. This problem becomes even more severe if the chromophore is not in its electronic ground state. A possible solution is to reparametrize the MM force field for the chromophore using accurate quantum chemistry calculations as a reference.^21−23^

A different strategy consists in using QM/MM MD simulations to achieve a correct description of the potential energy surfaces of the embedded QM subsystem. Such an approach is obviously computationally much more demanding, as it requires many QM/MM MD simulations to be performed. The latter are expensive, and only a limited time window can be simulated. However, they offer transferability and an overall very good accuracy. Moreover, they can be extended to excited states of chromophores within a Born–Oppenheimer approximation or in a nonadiabatic formulation when different adiabatic electronic states interact. In both cases, switching to an ES description results in a significant increase in computational cost. In this strategy, however, a delicate issue is the model used for describing the coupling between the QM and the MM subsystems. Commonly, this coupling is limited to electrostatic interactions. This clearly represents a limitation as the electric field produced by the MM subsystem is independent of the QM one and cannot respond to changes in the electronic density. This can become particularly relevant if an electronic excitation process has to be studied. In the past several decades, many polarizable embedding strategies have been developed to address this shortcoming (see ref (24) for a recent review, and the references therein).

In particular, our group has been focused on the development of a versatile and efficient polarizable QM/MM strategy^25−27^ based on density functional theory (DFT) and the highly accurate AMOEBA polarizable force field.^28,29^ Our implementation relies on a highly optimized linear-scaling machinery^30^ and has been successfully extended to Born–Oppenheimer MD (BOMD) simulations.^31,32^ In our implementation, QM/AMOEBA molecular dynamics is achieved through the interplay of the Tinker^33,34^ and Gaussian^35^ software packages. The Tinker MD package is used to compute the bonded and dispersion-repulsion contributions to the energy and forces and to propagate the MD trajectories. At the same time, the development version of the Gaussian suite of programs is used to compute the QM and polarizable embedding contributions to the energy and forces.

**Figure 1:**
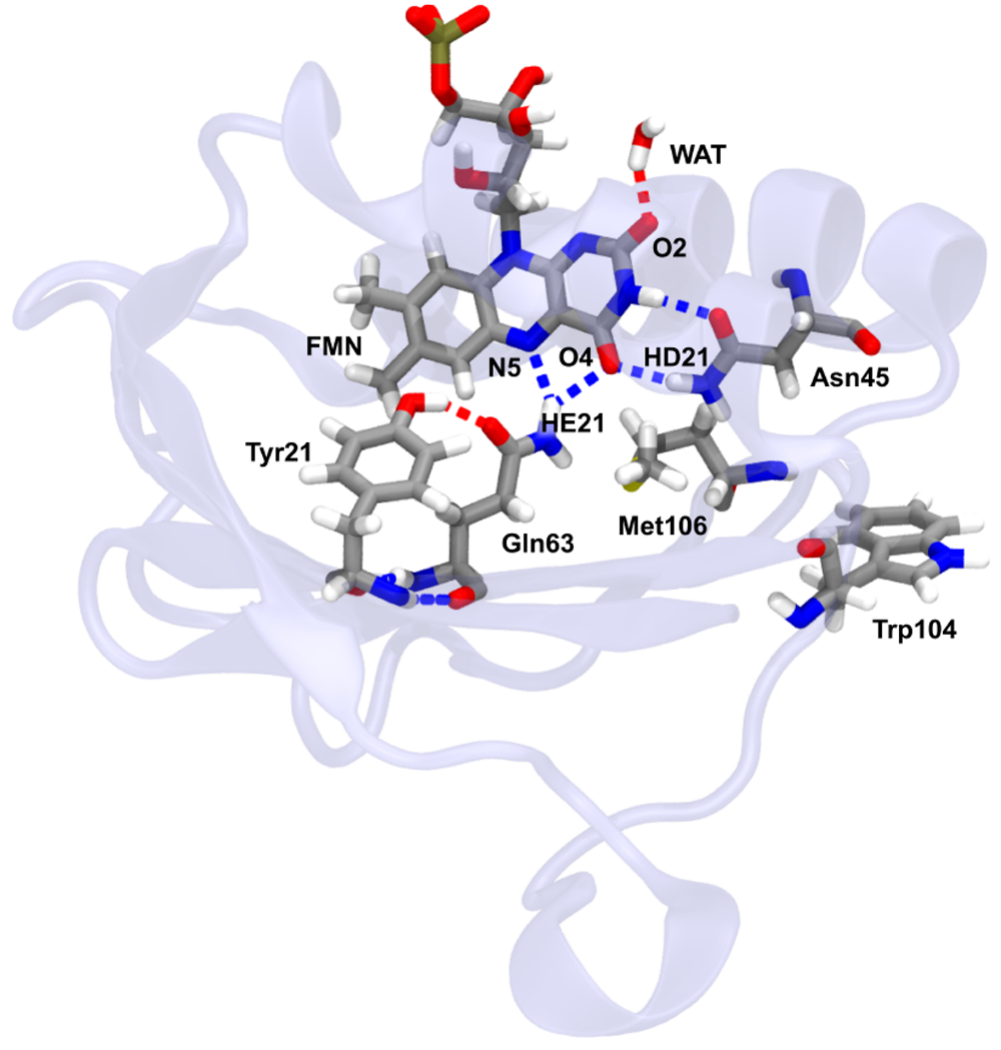
Flavin binding pocket of AppA in PDB: 2IYG.^39^ The chromophore, a flavin mononucleotide (FMN), is shown along with the interacting residues Tyr21, Gln63, and Asn45 as well as Met106 and Trp104. The protein backbone is shown in a semitransparent color.

In this contribution, we stretch the computational limits of our machinery by applying it to the study of the ultrafast vibrational spectroscopy of a blue-light-utilizing flavin (BLUF) protein found in bacteria, the AppA antirepressor.^36^ In the active site of AppA, the chromophore is noncovalently bonded to the protein, with hydrogen bond interactions formed with several amino acid residues (Figure 1). The photoactivation process of AppA has been largely investigated from both the experimental and the computational point of view.^8,9,37−54^ Among the different experimental techniques that have been used, we focus here on the time-resolved infrared (TRIR) difference spectra, which are sensitive to structural changes in both the chromophore and the surrounding protein.

Our strategy consists in combining the protein conformational sampling achieved through MM MD with QM/AMOEBA BOMD simulations describing both the ground and excited electronic states of the chromophore. To the best of our knowledge, this is the first time that the simulation of IR properties of a conjugated system embedded in a protein is attempted using excited-state, polarizable BOMD simulations.

We demonstrate that ground-state (GS) and excited-state (ES) BOMD trajectories can be used to simulate transient IR spectra and gain insight into the ultrafast structural changes of a protein-embedded chromophore induced by the excitation. This is achieved by calculating the spectra through the autocorrelation dipole moment of the GS and ES trajectories and assigning the corresponding normal modes through a signal-processing technique. By reproducing the transient IR spectrum of AppA, it was possible to assign the bands observed in the measured difference spectrum. In particular, we could characterize the flavin isoalloxazine-ring region of the spectrum, for which a full and clear description was missing.

## 2 Methods

### 2.1 Molecular Dynamics

Classical molecular dynamics (MD) simulations for AppA, in the so-called Met_*in*_ (PDB ID: 2IYG)^39^ and Trp_*in*_ (PDB ID: 1YRX)^37^ configurations, were recently reported by us.^54^ In the same study, starting from configurations extracted from the classical MD, we also reported QM/AMOEBA simulations for the electronic ground state of the embedded chromophore, a flavin mononucleotide (FMN), using the Gaussian/Tinker interface previously described. The QM/AMOEBA MD simulations were started from QM/AMOEBA optimized geometries and classically propagated for 10 ps.

We observed higher stability and better spectroscopic agreement with the experiments of the Met_*in*_ conformation over the Trp_*in*_ one. Thus, in the present study, we extend 10 QM/AMOEBA simulations of AppA, in the Met_*in*_ conformation, to the first electronic excited state of FMN. The choice of the QM/MM interface was identical to the ground-state trajectories.^54^ The isoalloxazine ring of the FMN, including the C1′ atom, was treated at the QM level, whereas the MM part included the ribityl tail, the entire protein, and a solvent (water and ions) shell of 30 Å around the chromophore. A link-atom strategy was used to describe the bonded interface between the QM and MM parts. The ribityl tail was cut at the C1′–C2′ bond. The charges, multipoles, and polarizabilities of the C2′ atom and its hydrogen neighbor were zeroed. The removed charge was redistributed on the nearest MM heavy atom neighbors.

Initial configurations and momenta for the electronic excited-state MDs were extracted from ground-state trajectories exactly at the 5 ps time mark. In this way, for each initial configuration, we obtained a ground-state and an excited-state trajectory lasting 5 ps with the same initial conditions.

For both ground- and excited-state trajectories, the QM part was described at the DFT ωB97X-D*/*6-31G(d) QM level, while the rest of the system was represented by the AMOEBA force field. The choice of the QM level was dictated by the need of a qualitatively correct description of ground and excited states, while at the same time limiting the computational cost. The long-range corrected ωB97X-D functional was chosen to avoid artificial mixing of the S_1_ state, as found in our previous work on another flavoprotein.^55^ This is extremely important in order to avoid an unphysical description of the excited-state potential energy surface.

To include nonperiodic boundaries, all of the residues beyond 22 Å from the FMN have been frozen, and to speed up the calculations, all of their AMOEBA polarizabilities were set to zero. Simulations were propagated for 10 ps (ground state) and 5 ps (excited state) in the NVT ensemble, using an integration step of 0.5 fs and the Velocity Verlet algorithm. A constant temperature of 300 K was kept using the Bussi thermostat,^56^ with a time constant of 0.1 ps.

### 2.2 Infrared Vibrational Spectra Calculation

Infrared vibrational absorption spectra can be calculated by Fourier-transforming the dipole moment autocorrelation function:^57^
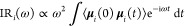
1where ⟨**μ**_*i*_(τ) **μ**_*i*_(τ + *t*)⟩_τ_ is the autocorrelation function of the molecular dipole on the Born–Oppenheimer trajectory of state *i*, that is, either the ground or the excited state. An equivalent expression can be obtained in terms of the time derivative of the dipole.^57^ The average ⟨·⟩_τ_ is performed, in principle, at all times τ of an infinite trajectory. In practice, the trajectory time is limited by the length of the simulation. In order to avoid “border” effects in the Fourier transform, the autocorrelation function is damped with a decreasing function to ensure its smooth decay to zero within the finite simulation time. Here, we use an exponential function with time constant 1.2 ps. The dipole was computed as the integral of the dipole moment operator with the appropriate GS or ES density. For the GS, we used the DFT self-consistent field density, whereas for the ES we used the relaxed TDDFT density.^58^

The first 5 ps of the ground-state trajectories were considered an equilibration of the system in the new QM/AMOEBA potential energy surface and were discarded for our calculations. Therefore, structural analyses and vibrational spectra for all ground- and excited-state trajectories were calculated each for 5 ps of simulation time (corresponding to 10 000 frames spaced 0.5 fs).

### 2.3 Normal-Mode Assignment Based on SOBI Analysis

The full vibrational density of states (VDOS) of the system can be obtained from the power spectra of all atomic velocities.^57^ The contribution of a single atom to the VDOS can be computed as the Fourier transform of the velocity autocorrelation function of that atom. For simple systems, effective normal modes can be obtained as the combination of Cartesian coordinates that minimizes the spread of frequencies of the corresponding power spectrum.^59^ An alternative way of assigning normal modes is to investigate the power spectra of internal coordinates, such as bond lengths and angles.^57,60^ Recently, a graph theory framework based on internal coordinates was proposed to aid mode assignment.^61^ Here, we apply a technique used in the signal-processing community, called second-order blind identification (SOBI),^62^ to find combinations of coordinates that oscillate independently, approximating the effective normal modes of our trajectories. The starting point of our transformation is represented by the internal coordinates of the QM part *x*_*i*_(*t*). Each of these coordinates is assumed to evolve in time as a combination of independent signals *s*_*j*_(*t*), oscillating at different frequencies. These independent components represent the effective normal modes of the system and can be written as
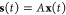
2The cross-correlation function of independent signals, ⟨*s*_*i*_(τ) *s*_*j*_(τ + *t*)⟩_τ_, should approach zero for *i* ≠ *j*. However, due to the finite nature of the trajectory, this relation holds only approximately. We seek the transformation *A* that gives the “best” independent coordinates. To do so, the internal coordinates are first standardized (i.e., their mean is subtracted, and they are divided by their standard deviation). Then, the time-lagged correlation matrices
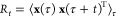
3are built for several lag times *t*. For *t* = 0, *R*_*t*_ represents the correlation matrix of the internal coordinates. The time-lagged correlation matrix of the independent components, *R̃*_*t*_, reads
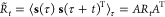
4In order to minimize the cross-correlation among different *s*_*j*_(*t*), the transformation matrix *A* should diagonalize *all* matrices *R*_*t*_. In practice, a joint diagonalization algorithm^63^ is used to ensure that the matrices *AR*_*t*_*A*^T^ are approximately diagonal for several lag times *t*.

As starting internal coordinates, we use all of the bonds within the conjugated part of the isoalloxazine ring, plus all the angles of the ring that involve hydrogen atoms. From each of the GS trajectories, we compute the time-lagged correlation matrices for equally spaced lag times (*t* = 50, 100, ..., 500 fs). For each lag time, we average the correlation matrix over all trajectories, in order to obtain a single transformation matrix *A*_GS_. The same procedure is repeated for the ES trajectories, and a different transformation matrix *A*_ES_ is obtained.

## 3 Results and Discussion

### 3.1 Excited-State Evolution of the Chromophore

We begin by analyzing the short-time evolution of FMN upon excitation.

The excitation process was simulated by taking nuclear coordinates and momenta from the GS trajectory and instantaneously switching the potential energy to that of the ES. Physically, this corresponds to exciting the system with an ultrafast pulse in resonance with the S_0_–S_1_ energy gap.

**Figure 2:**
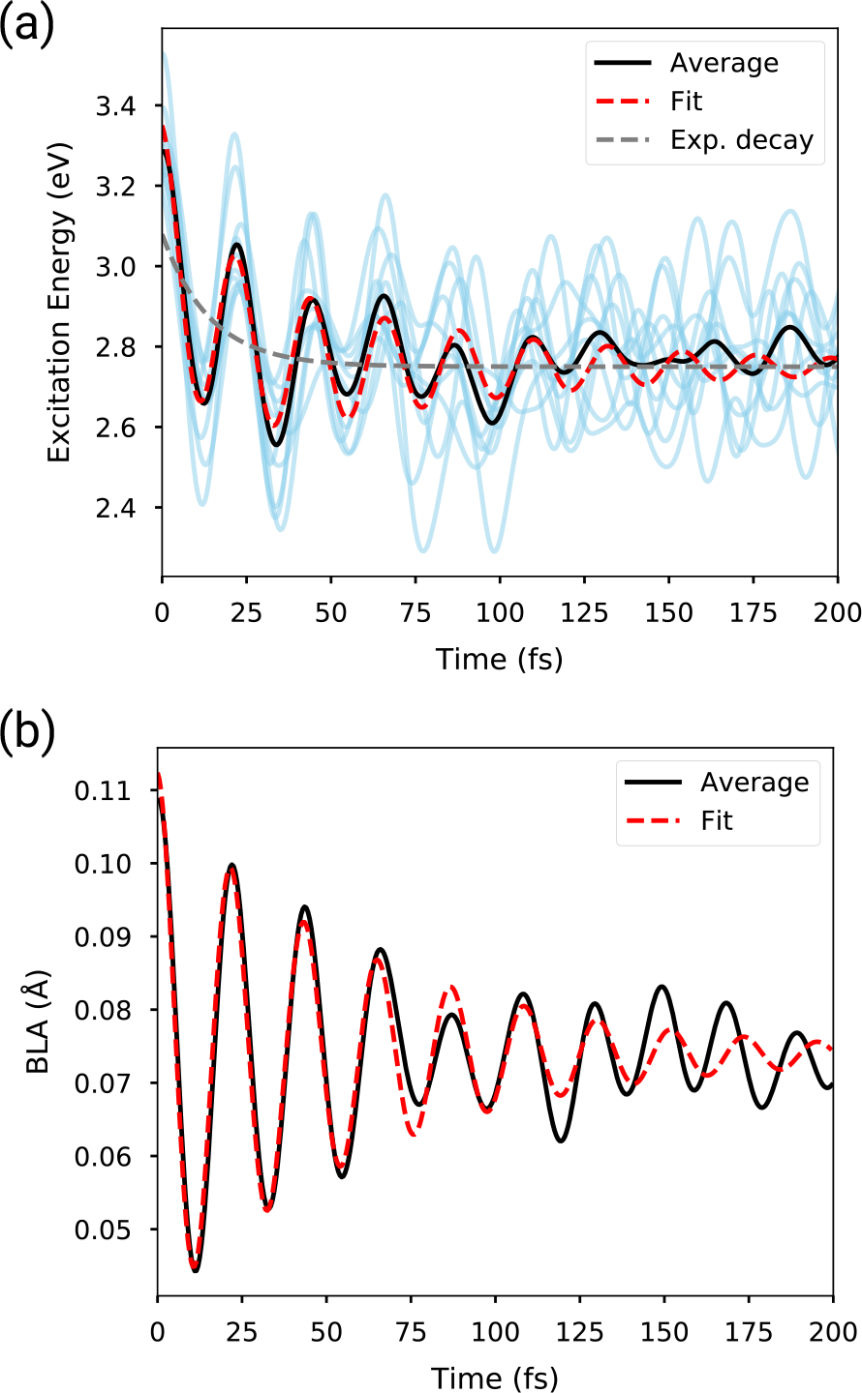
(a) Evolution of FMN energy gap calculated on the ES trajectory along the first 200 fs following excitation. The semitransparent blue lines show the evolution of individual trajectories, whose average is represented by the black line. The red dashed line is a fit with eq 5, and the gray line represents only the first and constant term of eq 5. (b) Evolution of the bond length alternation in the first 200 fs. Only the average is shown along with the fit using eq 5. Here, the exponential relaxation amplitude *A*_rel_ is negligible.

In Figure 2a, we show the time dependence of the ES–GS energy gap in the first 200 fs following excitation, calculated for all 10 ES QM/MM trajectories, along with their average. The first transient is characterized by high-amplitude oscillations common to all trajectories, but after ∼100 fs, each trajectory follows a different phase. The average energy gap shows large-amplitude oscillations, which are quickly damped by the dephasing between different trajectories. The analysis of the average allows capturing a slower decreasing trend of the energy gap, which reduces from the initial ∼3.3 eV to less than 2.8 eV within the first 100 fs.

In order to disentangle the oscillations from the slower relaxation, we fitted the time trace of the energy gap with the sum of a damped oscillation and exponential relaxation:
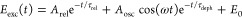
5with parameters τ_rel_, τ_deph_, ω (time scales of relaxation and damping, and oscillation frequency), *A*_rel/osc_ (amplitude of relaxation/oscillation), and *E*_0_ which is a constant offset. *E*_exc_(*t*) is the time-dependent energy gap, averaged on all the trajectories. The exponential relaxation (Figure 2, gray dashed line) is complete in less than 100 fs, suggesting that the dynamic Stokes shift in this system is ultrafast and mainly driven by the internal reorganization of the chromophore. This internal reorganization corresponds to a loss of potential energy, which is transferred from the chromophore to the surroundings.

The oscillations have a period of ∼22 fs, corresponding to a frequency of 1515 cm^–1^. These oscillations correspond to the C=C and C=N stretching modes of the isoalloxazine ring. This is confirmed in Figure 2b, where we report the time evolution of the bond-length alternation (BLA) for all single and double bonds of the isoalloxazine ring. In fact, these oscillations have the same oscillation period (thus, the same frequency) of the aforementioned energy gap. However, in the BLA, we cannot distinguish any exponential relaxation, i.e., the first term of eq 5. The BLA, which oscillates around 0.11 Å in the ground state, relaxes as an underdamped oscillator toward the excited-state average of ∼0.08 Å. This relaxation is a consequence of the change in equilibrium values for the ring bond lengths upon excitation from GS to ES.

**Figure 3:**
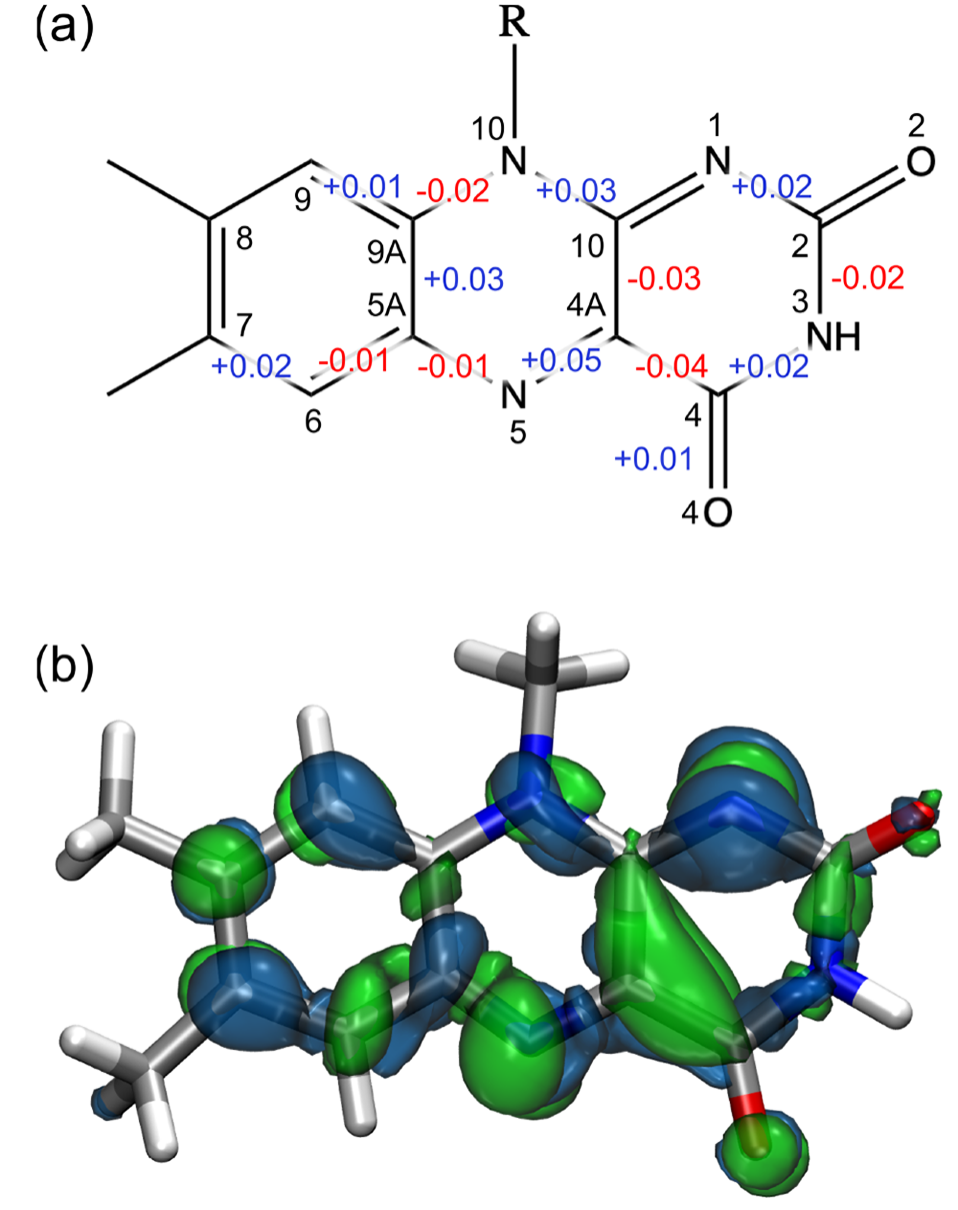
(a) Structure formula of the flavin isoalloxazine-ring with average bond length differences (excited-minus-ground) in the QM/MM MDs. Positive and negative bond length differences are shown in blue and red, respectively. Values below 0.01 Å are not shown. (b) Density difference (excited-minus-ground) of flavin in AppA. Positive (negative) values are shown in green (blue). Isovalues are ±1.5 × 10^–3^ atomic units.

In order to investigate the change in bond lengths upon excitation, we calculated the average bond length for all bonds of the ring during the excited-state trajectories as well as on the ground-state trajectories starting from the same initial conditions. The corresponding change is shown in Figure 3a. As expected, upon excitation, almost all conjugated bonds change their equilibrium distance. However, the greatest bond length reorganization occurs around the C4A atom: the C4A=N5 double bond lengthens significantly, and the adjacent C4—C4A bond shortens by roughly the same quantity.

The alternation between single and double bonds is generally preserved upon excitation; i.e., there is no inversion of bond lengths. Interestingly, the C9A–C5A bond, which has some single-bond character in the ground state, becomes even longer in the excited state, indicating that the bond length reorganization is quite complex in this chromophore.

The change in bond lengths upon excitation can be rationalized by looking at the natural transition orbitals of the GS → ES transition and at the density difference upon excitation (Figure 3b). A density increase can be easily seen between atoms C4, C4A, and C10, whereas a density depletion is present on the side of C9 and C9A. This explains the significant shortening of both C4–C4A and C4A–C10 bonds. In addition, we can notice an increase in the density on the O4 carbonyl oxygen, and especially on the N5 atom.

**Figure 4:**
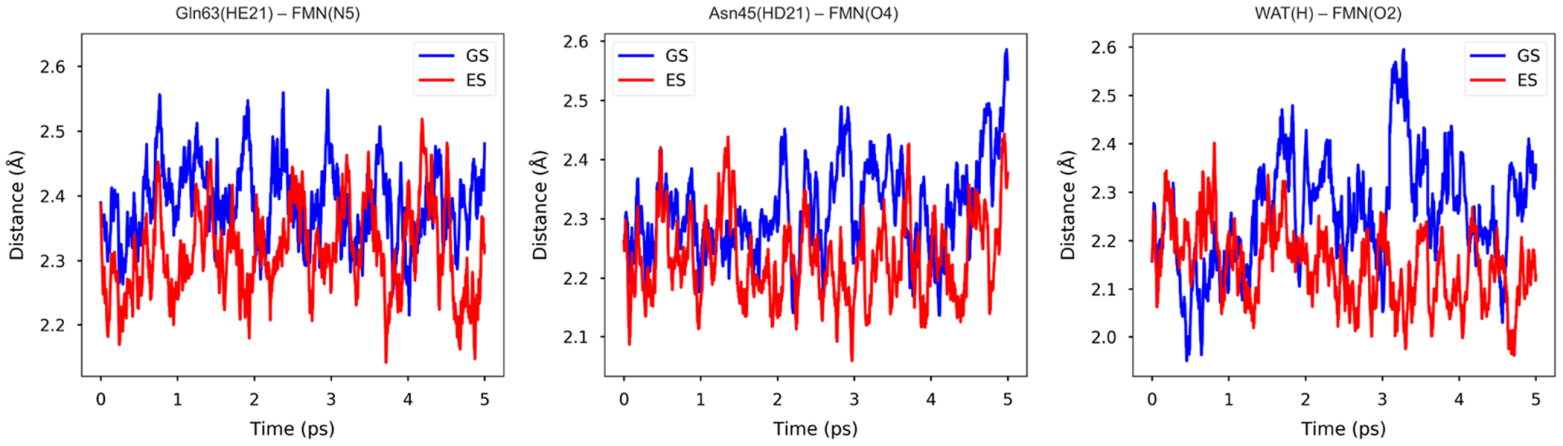
Time evolution of hydrogen bond interactions of flavin with key protein residues and water. In all plots, we report for all times the atom–atom distance averaged over the 10 trajectories of the ground state (blue) and excited state (red). In the case of the hydrogen bond with water (right), at each time, we computed the minimum distance between the O2 atom and the water hydrogen atoms; then, we took the average over the trajectories.

An electronic redistribution can also have an effect on the interaction with residues which are directly involved in the hydrogen bonds with the flavin (see Figure 1). We have compared intermolecular hydrogen-bond interactions between GS and ES trajectories. In particular, we focused on Gln63(HE21)-FMN(N5), Asn45(HD21)-FMN(O4), and WAT(H)-FMN(O2) hydrogen bonds (Figure 4), which all seem to become tighter in excited-state trajectories, although the differences are not very large. We calculated the time evolution of the atom–atom distance for each H-bond averaged over the 10 trajectories for each GS and ES state. The most substantial effect is found for the H-bond between the Gln63 amide hydrogen and the N5 atom, which becomes shorter within the first 100 fs after the excitation and oscillates at around 2.3 Å. In the GS trajectory, for comparison, this H-bond is more elongated and oscillates around 2.4 Å. A distinct change in the fluctuation of the H-bond between water and O2 of FMN is observed after 3 ps (Figure 4, right). By plotting the single plot for each GS trajectory, it is noticed that two of the trajectories exhibit significantly large fluctuations (Figure S1) that lead to this distinct change in the average plot.

Having investigated the ultrafast response of the chromophore to electronic excitation, we now move to the analysis of vibrational properties.

### 3.2 IR Spectra and Normal-Mode Analysis

We computed IR spectra from the dipole–dipole autocorrelation function on both ground-state and excited-state QM/AMOEBA trajectories, as described in Section 2.2. The results for all trajectories are shown in Figure S2 of the SI.

The ground-state spectra present a band in the carbonyl region at ∼1800 cm^–1^, which in some trajectories is split into two peaks. Its frequency is blue-shifted with respect to the experiments^8,9^ due to a systematic error of the chosen level of theory (see Table S1 in the SI). However, for the sake of clarity, we refrain from applying a scaling factor to the frequencies, which would complicate the analysis at this point. Two further peaks appear at 1600–1700 cm^–1^, which are ascribed to the isoalloxazine-ring modes and will be analyzed below.

The IR spectra of all GS trajectories were averaged for comparison with the measured absorption spectrum of FMN in water (see Figure S3 in the SI).^64^ All bands are blue-shifted and more intense in the measured spectrum. However, considering the different environment in which the IR spectrum of FMN is calculated or measured (in a protein-matrix or in water, respectively), the agreement is good. The isoalloxazine-ring region appears as composed by two main signals in both spectra, while the carbonyl region bands are broader in the experiment due to the formation of hydrogen bonds with the aqueous solvent.

Now, we can compare the ES spectra to the GS ones (Figure S2 of the SI), and we immediately notice a red-shift of the carbonyl stretching band from about 1820 to about 1780 cm^–1^, a loss in intensity of peaks in the 1600–1700 cm^–1^, which has been assigned to the isoalloxazine-ring normal modes in the literature,^8,9,65^ and the appearance of intense peaks in the 1400–1450 cm^–1^ region. The excited-state peaks were tentatively assigned to the conjugated ring.^8^

Assigning these peaks to normal modes is a complex task, owing to the large number of degrees of freedom, to the mode delocalization induced by the extended conjugation, and to the overlap between different signals in the computed IR spectrum. In our previous study,^54^ we showed that it is possible to apply a signal-processing technique, called SOBI (see the Methods section), to differentiate between the C_2_=O and C_4_=O stretching signals for the ground-state trajectories (see Figure 3a for atom numbering). We start by applying the SOBI technique on both carbonyl and isoalloxazine-ring stretching regions (range 1400–1900 cm^–1^) on the ground-state QM/AMOEBA trajectories. The power spectra of SOBI coordinates are well localized in frequency (Figure S4), demonstrating the ability of this technique to find meaningful normal modes.

Conversely, the power spectra obtained directly from the internal coordinates are spread in a wide range of frequencies (see Figure S5). For example, the C8–C9 bond shows peaks at both ∼1700 and 1400 cm^–1^, and several bonds oscillate at 1600 cm^–1^. The normal-mode SOBI power spectra, instead, are able to distinguish even the two overlapping peaks around 1600 cm^–1^.

As explained in Section 2.2, SOBI coordinates are obtained averaging the time-lagged correlation matrices over all trajectories (Figure S4, upper panel). However, we have previously shown (see Section 3.1) that two GS trajectories exhibit larger fluctuations for the H-bond distance between water and O2 of FMN. Therefore, to verify the robustness of our strategy, we have recomputed a new set of SOBI coordinates excluding the two GS outlier trajectories. Thus, we have recalculated the GS power spectra of these coordinates (Figure S4, lower panel). As we can see, the signals in the carbonyl and isoalloxazine-ring stretching regions are almost all identical.

**Figure 5:**
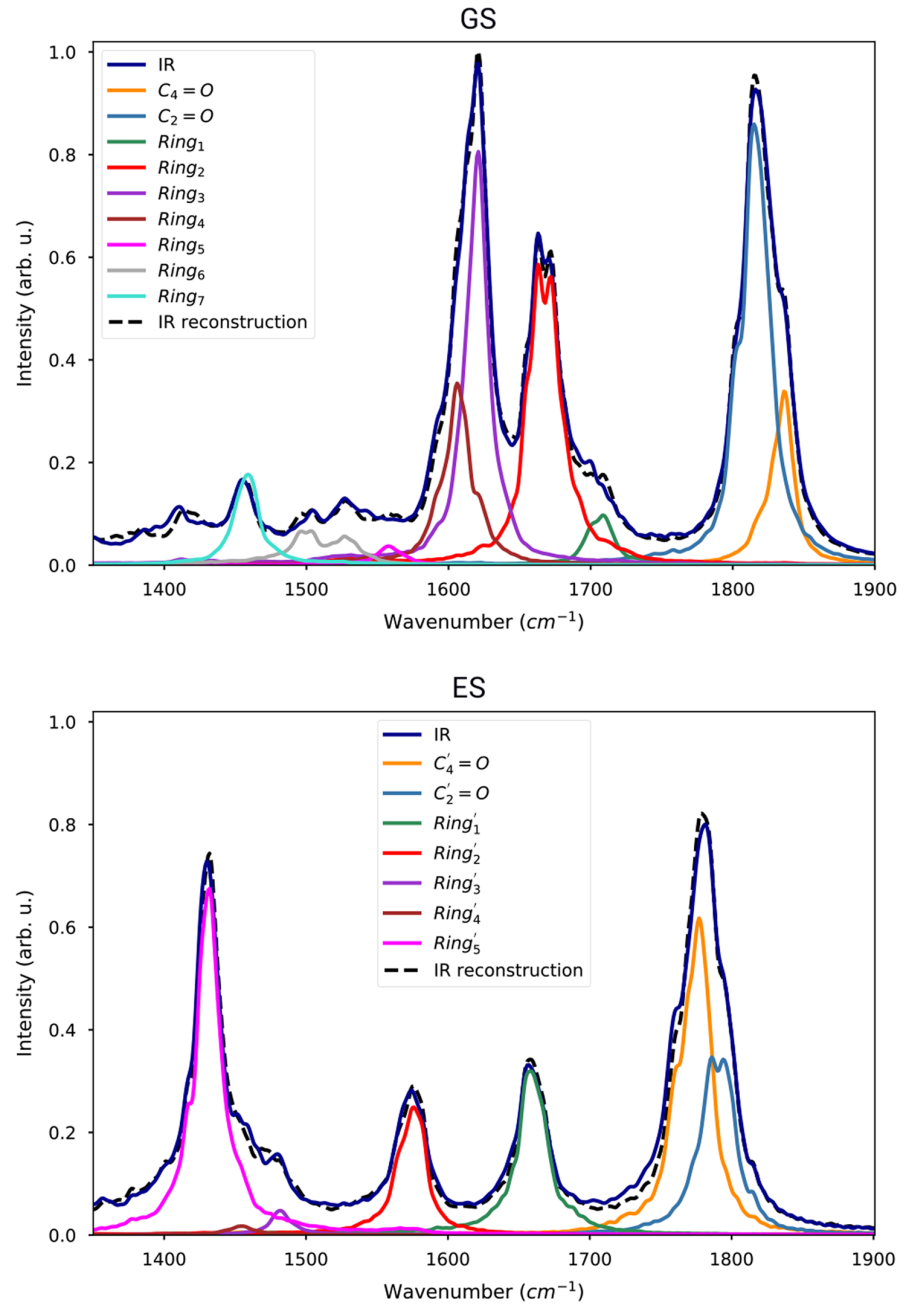
Average IR spectra of GS (upper panel) and ES (lower panel) fitted with a combination of the power spectra of SOBI coordinates. The average IR spectrum is represented by the black solid line. The IR spectrum reconstructed as a linear combination of SOBI internal-coordinate power spectra is shown as a dashed line. The individual contributions are shown as colored lines.

To show that the obtained mode coordinates can be used to assign the IR peaks, we fitted the GS average IR spectrum with a linear combination of mode power spectra. In Figure 5, we show that the IR spectrum in the ring and carbonyl regions can be reconstructed from these combinations. This procedure allows us to easily assign the IR spectra even in the case of overlapping signals. Six ring modes can be found in the region above ∼1500 cm^–1^, denoted as *Ring*_1_ ... *Ring*_6_. Among these modes, *Ring*_2_, *Ring*_3_, and *Ring*_4_ contribute to the most intense peaks of the GS spectrum.

**Figure 6:**
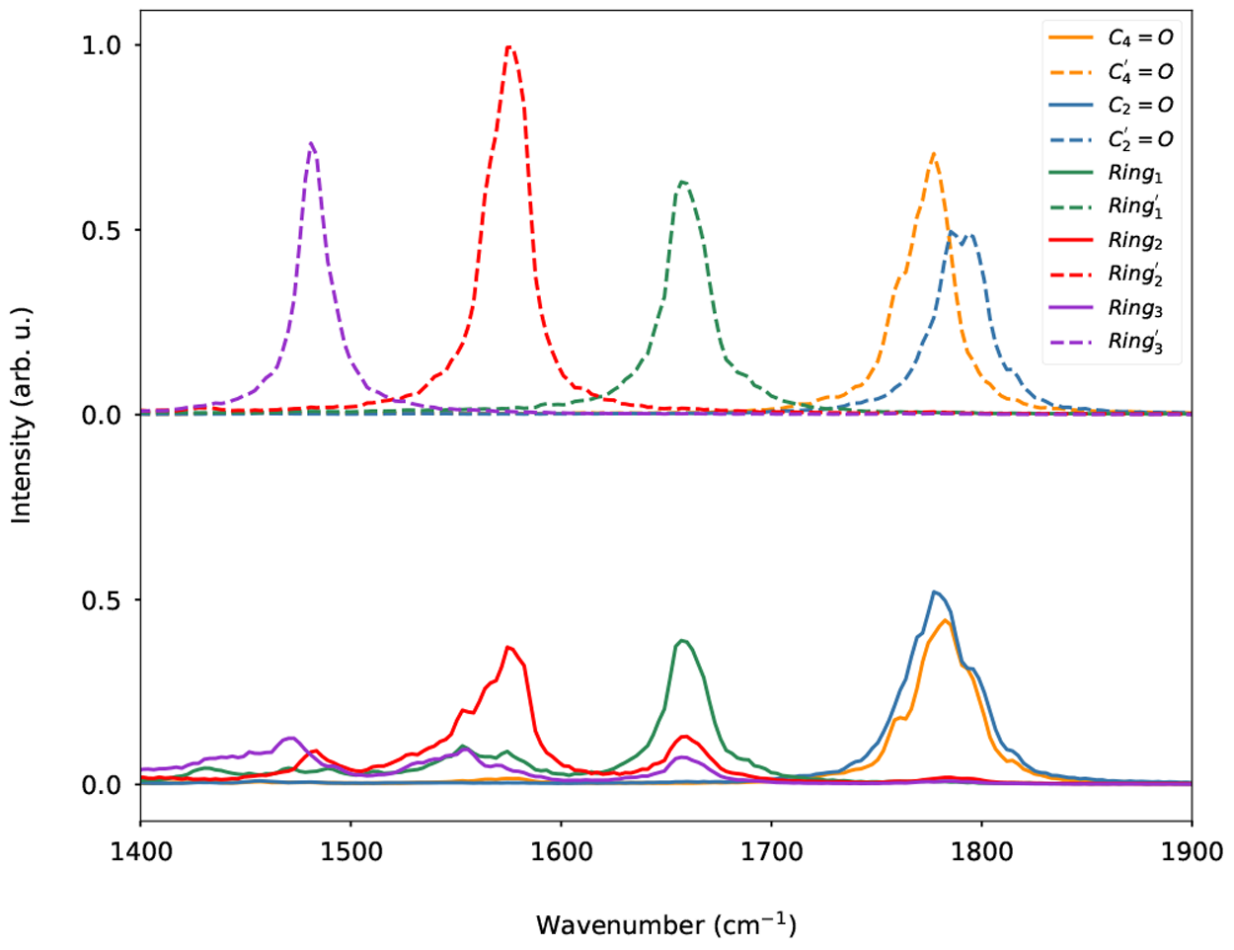
Average power spectra of the GS (continuous line) or ES SOBI coordinates (dashed line) calculated on the ES trajectories. The isoalloxazine-ring SOBI coordinates are numbered in decreasing frequency order.

We now move to the analysis of excited-state IR spectra. There are two ways of assigning excited-state IR signals. Assuming that the normal modes of ground and excited states are similar, it is possible to compute the excited-state power spectra using the same coordinate transformation computed on the ground state. Alternatively, a new different transformation can be computed using the excited-state trajectories, obtaining normal modes specific to the excited state. Using the ground-state modes for both trajectories would simplify the assignment of IR peaks. However, we found that applying the ground-state transformation did not result in well-separated signals (Figure 6, lower panel). On the other hand, the excited-state transformation could again recover power spectra localized in frequency and well separated (Figure 6, upper panel). Finally, we have used the power spectra of SOBI coordinates to fit the ES average IR spectrum: the comparison between the fit and the calculated spectra, reported in Figure 5, shows a very good agreement confirming once again the validity of the state-specific SOBI coordinates in reproducing the IR spectra.

**Figure 7:**
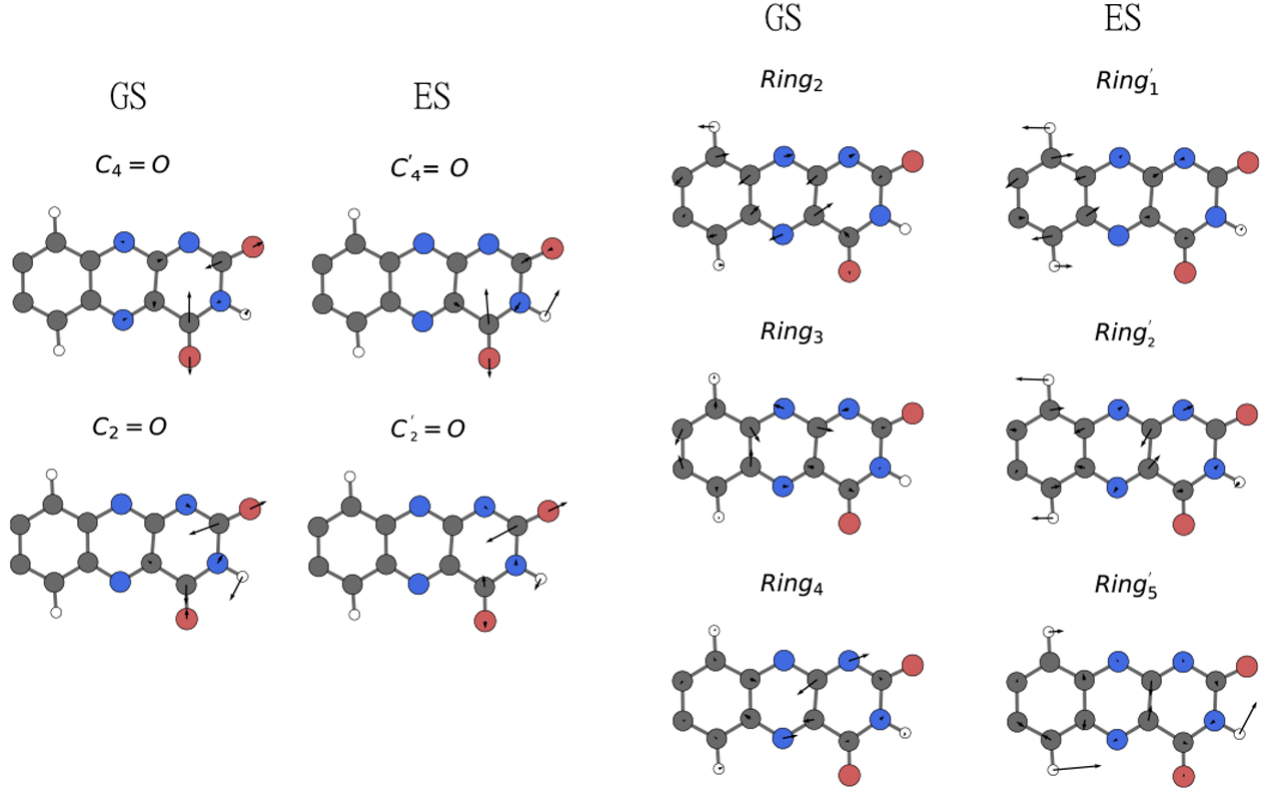
Graphical representation of (left panel) carbonyl normal modes and (right panel) isoalloxazine-ring normal modes calculated on the GS and ES trajectories.

Once the peaks are assigned to normal modes, we can also examine their composition in terms of internal coordinates (see atom numbering in Figure 3a). According to experiments and harmonic calculations,^8^ the C_4_=O stretching mode is actually a symmetric combination (**C**_4_=**O** + C_2_=O), while C_2_=O is its asymmetric counterpart (**C**_4_=**O** – C_2_=O). Our analysis confirms this picture (Figure 7, left panel; and Figure S6), showing that the mode with the largest C_2_=O contribution has a contribution from C_4_=O with the opposite sign. The mode with the largest C_4_=O contribution is mixed with a C_2_=O contribution having the same sign.

In the excited state, however, the situation is reversed: C_4_=O becomes an asymmetric stretching and C_2_=O a symmetric one. This can be explained considering that, upon excitation, the C_4_=O bond undergoes a more substantial elongation than the C_2_=O (see above). The differential effect on the two bonds clearly also influences the mixing of the two stretching modes. We also notice that C_2_=O modes are mixed *in phase* with the C2—N3—H3 bending in both GS and ES modes. Similarly, the C_4_=O stretching has an *in phase* contribution from the C4—N3—H3 bending. The two bending contributions are particularly marked for the asymmetric C=O stretching, that is, GS C_2_=O and ES C_4_=O.

The same analysis is performed for the most intense (IR-active) isoalloxazine-ring normal modes (see Figure 7, right panel; and Figure S7). As expected, upon excitation, we also observe a change in the nature of the ring normal modes. In the excited state, the SOBI ring modes are more delocalized, and there is a larger participation of bending modes.

**Figure 8:**
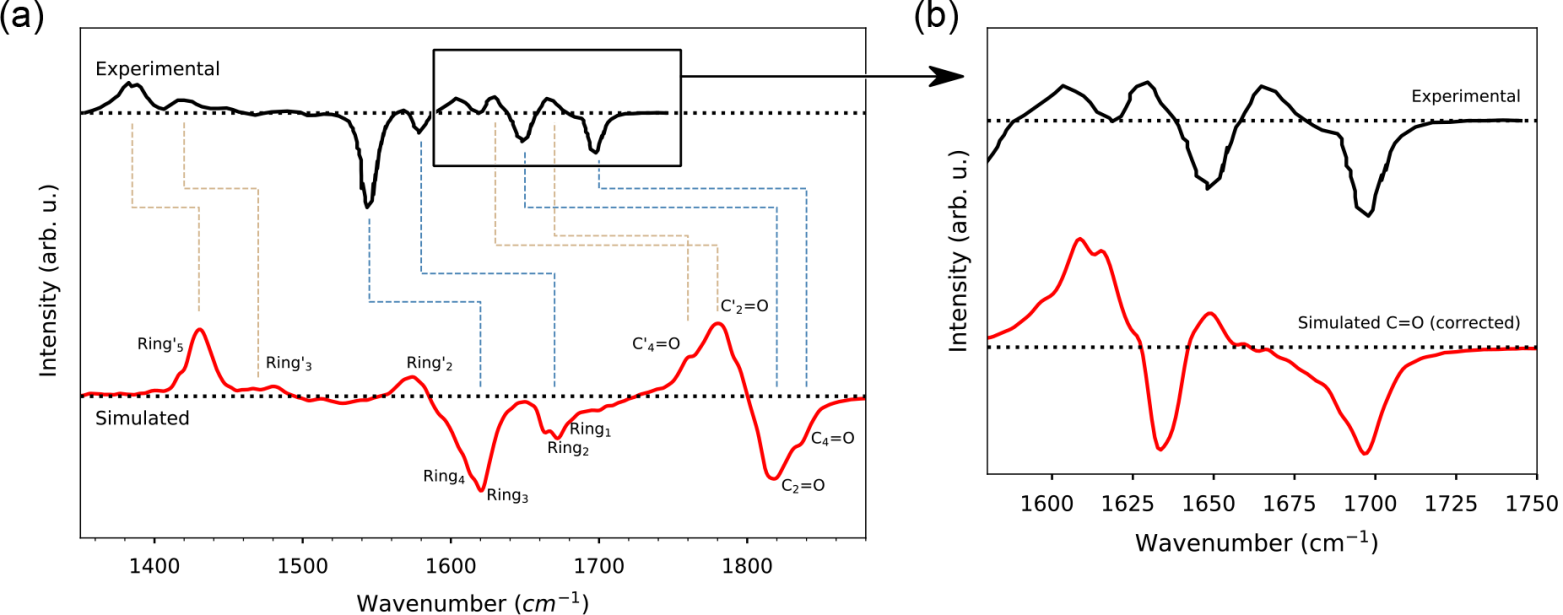
Comparison between measured^9^ and simulated TRIR difference spectra of AppA and assignment to normal modes. (a) The difference spectrum was obtained by computing the difference between the average (over the trajectories) ES and GS IR simulated spectra. Positive and negative peaks correspond to ES absorption and GS bleaching signals, respectively. The normal-mode assignment was performed on the basis of the presented SOBI analysis. The square box indicates the carbonyl region in the experiment. Excited-state normal modes are indicated with a prime (′) symbol. The calculated spectrum has not been scaled. (b) Difference spectra in the carbonyl region. GS and ES C_2_=O modes were scaled by 0.901, while the C_4_=O modes were scaled by 0.924. The scaling factors were chosen in order to reproduce the C_2_=O and C_4_=O shift in GS (negative peaks).

We are now in a position to simulate the TRIR spectrum and compare it to the experiment. To this end, we took the difference (excited-minus-ground) of the averaged IR spectra calculated for ground- and excited-state trajectories. The result is shown in Figure 8a along with its experimental counterpart.^9^

We first focus on the carbonyl region. Negative peaks at 1700 and 1650 cm^–1^ in the experiment correspond to C_4_=O (symmetric stretching) and C_2_=O (asymmetric stretching) ground-state modes. Both signals are red-shifted in the ES and thus appear as two separate positive signals. Upon excitation, the C_4_=O and C_2_=O are red-shifted by 32 and 19 cm^–1^, respectively.^8,9^ In the simulated spectrum, the GS carbonyl modes are closer in frequency than in the experiment and thus appear as a single band. However, our calculations give the two modes in the same order as in the experiment: C_4_=O is more blue-shifted than C_2_=O, and they are both red-shifted in the ES. Experimentally, a larger red-shift upon excitation is seen for the C_4_=O signal than for the C_2_=O. This cannot be confirmed directly from our simulations, due to the two carbonyl modes being too close in the GS. However, a closer inspection of the power spectra of these modes (Figure S8 in the SI) reveals that the red-shift of the C_4_=O upon excitation (60 cm^–1^) is larger than the shift for the C_2_=O mode (25 cm^–1^). The problem of the relative position of the carbonyl modes in the calculated spectra may be solved by employing a larger basis set in the QM/AMOEBA BOMD simulations, as suggested by harmonic calculations *in vacuo* (Table S1 in the SI). To confirm this hypothesis without the need of repeating the already high-cost QM/AMOEBA BOMD simulations, we have reconstructed the carbonyl part of the spectrum by differently scaling the GS frequencies of the two carbonyl modes in order to separate their signals. The results are reported in Figure 8b and clearly show that now the main features of the experiments are all reproduced.

Then, close to the carbonyl region, at 1600 cm^–1^, we found a positive signal, which was not previously assigned but that we can associate to the excited-state *Ring*_1_^′^. This mode, unfortunately, is canceled out by the lower-frequency ground-state bleaching in the simulated difference spectrum.

Moving to the lower-frequency regions, the weaker bleaching at 1580 cm^–1^ and the very intense one at 1547 cm^–1^ in the experiment were assigned to isoalloxazine-ring modes, in particular to C4A–N5 and C10A–N1, respectively.^8^ Similarly, we can assign the calculated weaker bleach at 1760 cm^–1^ to the *Ring*_2_ mode (and, with a smaller contribution, to *Ring*_1_); the intense one at 1625 cm^–1^ is due to both *Ring*_3_ and *Ring*_4_ (located at 1600 cm^–1^) modes. As a matter of fact, and as shown in Figure S4, the largest contribution to *Ring*_2_ comes from C4A–N5 stretching, and the *Ring*_4_ mode is quite localized on C10–N1. Instead, *Ring*_3_ is quite delocalized on the entire isoalloxazine ring.

The measured positive signals at lower frequencies, 1440 and 1383 cm^–1^, were only associated with ring modes but not assigned to a specific one.^8^ However, the weak band at 1440 cm^–1^ was assigned to a weak transient triplet state.^9^ Here, we assign both positive bands to distinct ES ring modes, which are different from and more delocalized than the GS ones. In particular, the intense band at 1383 cm^–1^ is assigned to *Ring*_5_^′^, while the weak one at 1440 is assigned to *Ring*_3_^′^.

For completeness, in the simulated spectrum, we also observe a positive signal at 1580 cm^–1^, due to the *Ring*_2_^′^ ES mode. We cannot pinpoint this mode in the experiment, as it is probably canceled out by the intense bleaching at 1547 cm^–1^.

## 4 Conclusions

In this contribution, we show an effective way to simulate transient IR spectra of photoreceptors by combining long classical MM MDs (for the configurational sampling) with shorter QM/AMOEBA BOMD of both ground and excited electronic states. Within this framework, the spectra can be calculated by the autocorrelation dipole moment of the respective trajectories whereas normal mode assignment can be achieved by employing a signal-processing technique (SOBI). This integrated strategy has been applied to one of the most largely investigated BLUF proteins, AppA. We first analyzed the short-time evolution of the embedded flavin upon excitation noting that the initial transient (before ∼100 fs) is characterized by high-amplitude oscillations common to all trajectories corresponding to the C=C and C=N stretching modes of the isoalloxazine ring. A fit reveals an exponential relaxation which is complete in less than 100 fs, suggesting that the dynamic Stokes shift in this system is ultrafast and mainly driven by the internal reorganization of the chromophore. Moving to the IR spectra, we found that it is necessary to use a different normal-mode representation to describe either the ground- or excited-state normal modes. In this way, we could assign bands in the measured transient IR to specific normal modes. Remarkably, this strategy proved to be particularly efficient in the assignment of the flavin isoalloxazine-ring IR region, for which a full and clear description has not been reported.

We believe that the strategy here reported is general and may be applied to study other photoreceptors.

## Supporting Information Available

Harmonic carbonyl frequencies in a vacuum, GS and ES calculated IR vibrational spectra, power spectra of SOBI and local internal coordinates, and composition of SOBI normal modes (PDF)
