# Supplementary material for: Ultrafast Transient Infrared Spectroscopy of Photoreceptors with Polarizable QM/MM Dynamics
Source: J Phys Chem B. 2021 Sep 3;125(36):10282–92. doi: 10.1021/acs.jpcb.1c05753 (PMC8450903; doi:10.1021/acs.jpcb.1c05753)
Supplement: Supplementary file 1 — jp1c05753_si_001.pdf [file jp1c05753_si_001.pdf]

Supplementary Information: Ultrafast Transient Infrared Spectroscopy of Photoreceptors with Polarizable QM/MM Dynamics

Veronica Macaluso, Shaima Hashem, Michele Nottoli, Filippo Lipparini, Lorenzo Cupellini,* and Benedetta Mennucci*

Dipartimento di Chimica e Chimica Industriale, University of Pisa, via G. Moruzzi 13, 56124, Pisa, Italy

E-mail: lorenzo.cupellini@unipi.it; benedetta.mennucci@unipi.it

Supplementary Tables

Table S1: Harmonic C_2=O and C_4=O frequencies computed in vacuum on the isoalloxazine ring.

Functional	Basis set	freq$_{C_2=O}$ (cm^{-1})	freq$_{C_4=O}$ (cm^{-1})	freq$_{C_4=O}$ - freq$_{C_2=O}$ (cm^{-1})
B3LYP	6-31G(d)	1805.58	1814.55	7.97
B3LYP	6-311+G(d,p)	1760.26	1779.99	19.73
ωB97X-D	6-31G(d)	1864.19	1872.02	7.83
ωB97X-D	6-311+G(d,p)	1820.37	1838.44	18.07

Supplementary Figures

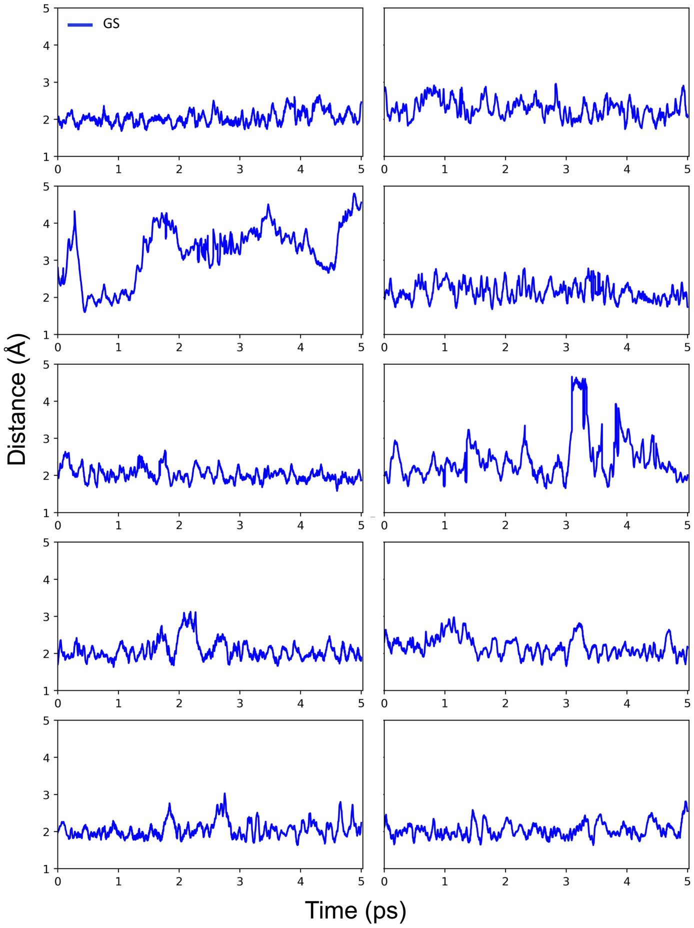

Figure S1: Time evolution of hyrogen bond distance between water and O2 of FMN in GS trajectories.

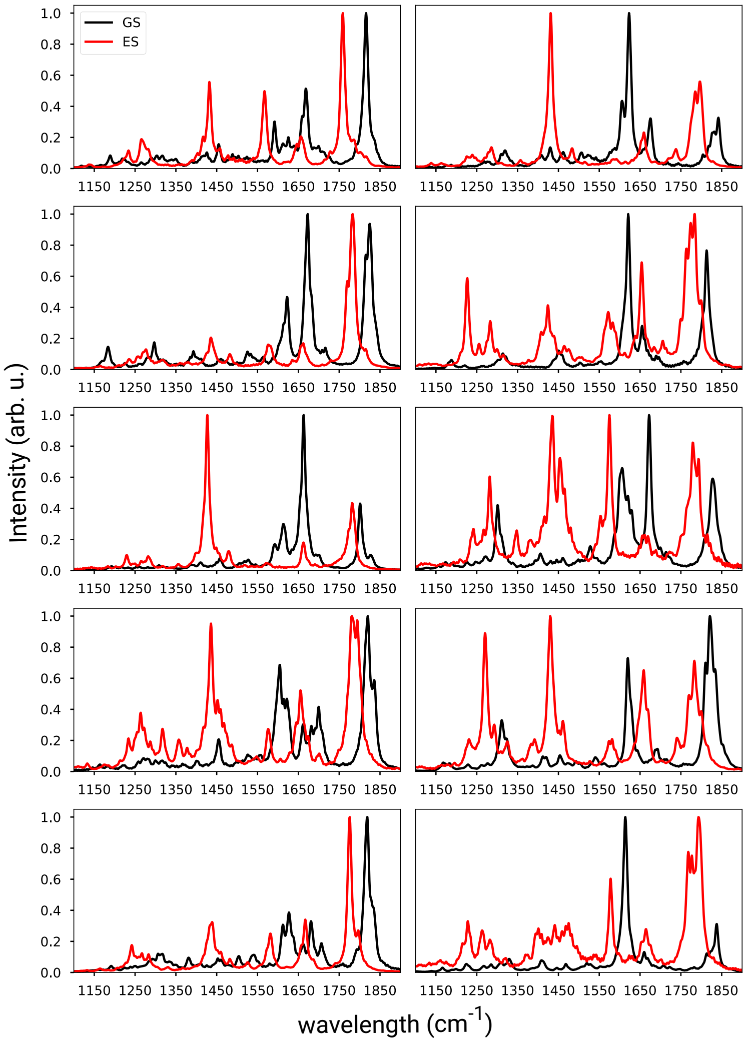

Figure S2: IR vibrational spectra calculated from GS (black) and ES (red) QM/AMOEBA dynamics. For GS, spectra were calculated only on the last 5 ps. All spectra were independently normalized. No scaling factor was applied.

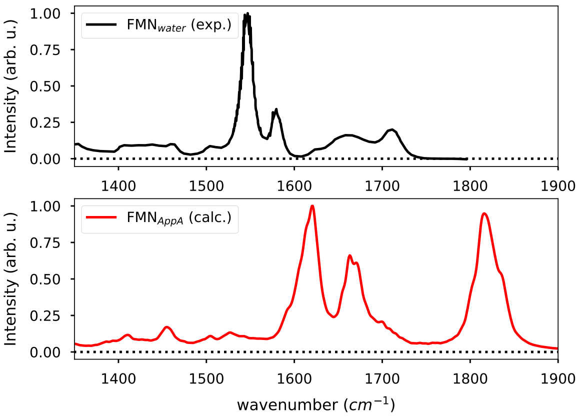

Figure S3: Comparison between computed (lower panel) and experimental (upper panel) IR absorption spectra of FMN. The experimental IR spectrum was measured in water.[1] The calculated spectrum was computed using QM/MMpol trajectories of FMN embedded in AppA protein matrix.

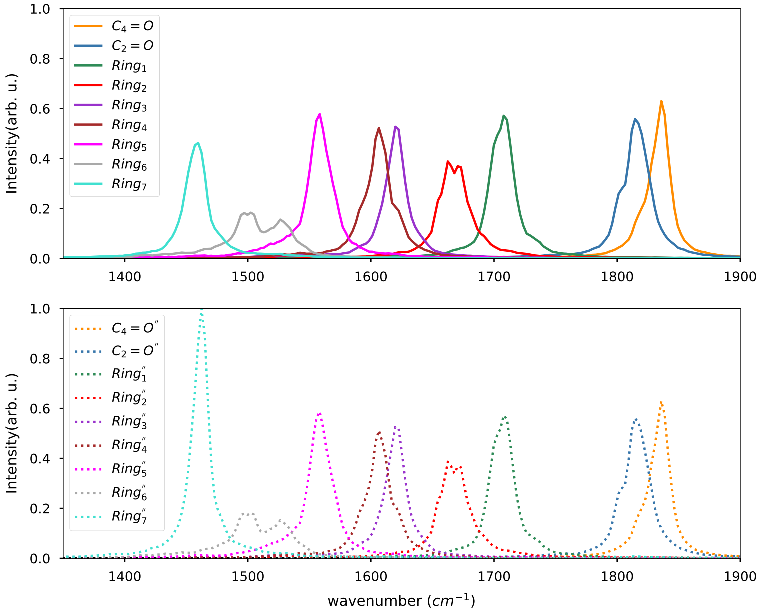

Figure S4: Average power spectra calculated over all GS trajectories of the SOBI coordinates. The SOBI coordinates were obtained averaging over all GS trajectories (continuous line, top panel) or excluding two out-layer trajectories (dashed line, bottom panel). Different colors refer to power spectra of various SOBI modes. Ring modes were numbered according to decreasing frequency order. Note that all analyses were performed using the power spectra/SOBI coordinates of the top panel.

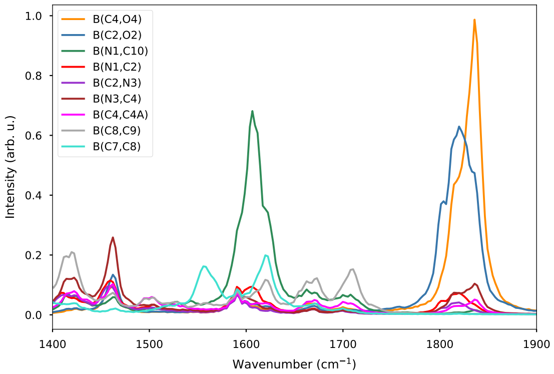

Figure S5: Average power spectra over all GS trajectories of localized isoalloxazine-ring internal coordinates.

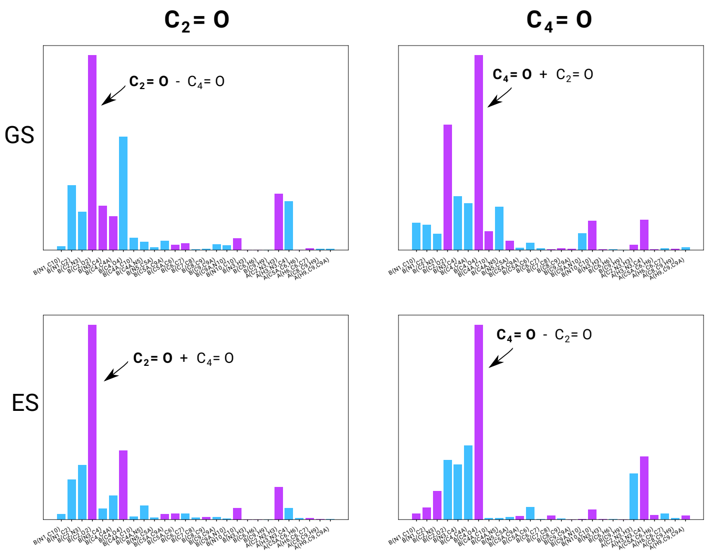

Figure S6: Composition of $\mathbf{C_4{=}O}$ and $\mathbf{C_2{=}O}$ modes for ground (GS) and excited (ES) states. $\mathbf{C_4{=}O}$ and $\mathbf{C_2{=}O}$ ICs are defined as those SOBI internal-coordinates to which $C_4{=}O$ and $C_2{=}O$ localized-bonds contribute the most, respectively. Different colors (light blue and violet) indicate an opposite-sign contribution to the mode.

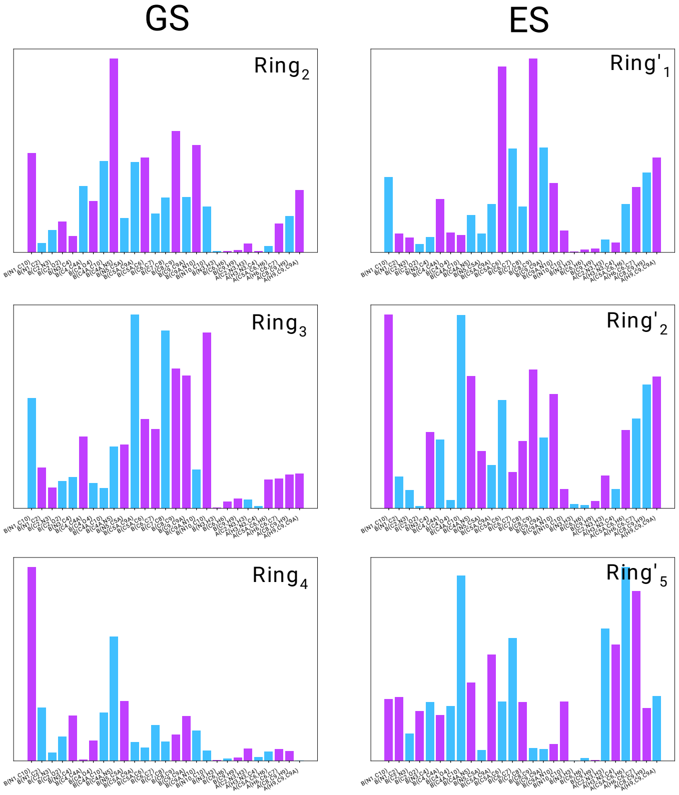

Figure S7: Composition of IR most-intense isoalloxazine ring-modes for GS and ES states. Ring-modes are numbered in frequency-decreasing order. Different colors (light blue and violet) indicate an opposite-sign contribution to the ICs.

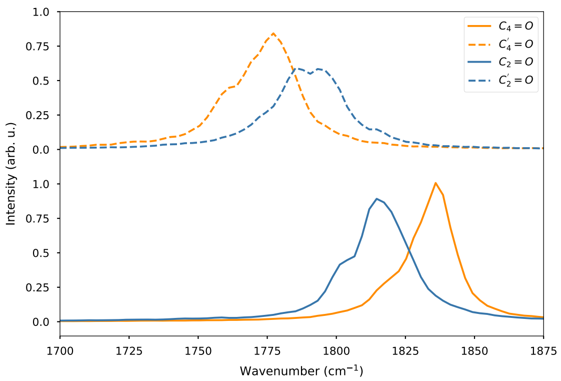

Figure S8: Average power spectra of Carbonyl SOBI modes computed on GS and ES trajectories. ES modes are labeled with a prime (') and represented as dotted lines.

References

(1) Spexard, M.; Immeln, D.; Thöing, C.; Kottke, T. Infrared spectrum and absorption coefficient of the cofactor flavin in water. *Vibrational Spectroscopy* **2011**, *57*, 282–287.
